# Hiccups in patients with cancer: a multi-site, single-institution study of etiology, severity, complications, interventions, and outcomes

**DOI:** 10.1186/s12885-022-09760-5

**Published:** 2022-06-15

**Authors:** Christopher J. Ehret, Yahya Almodallal, Jennifer G. Le-Rademacher, Nichole A. Martin, Michael R. Moynagh, Arush Rajotia, Aminah Jatoi

**Affiliations:** 1 Department of Oncology, Mayo Clinic, 200 First Street SW, Rochester, MN 55905 USA; 2 Division of Clinical Trials and Biostatistics, Mayo Clinic, 200 First Street SW, Rochester, MN 55905 USA; 3 Department of Radiology, Mayo Clinic, 200 First Street SW, Rochester, MN 55905 USA

**Keywords:** Hiccups, Morbidity, Complications, Palliation

## Abstract

**Background:**

To our knowledge, previous studies have not investigated hiccups in patients with cancer with detailed patient-level data with the goal of capturing a broad spectrum of hiccup symptomatology.

**Methods:**

This multi-site, single institution study examined consecutive medical records to better understand hiccups in patients with cancer.

**Results:**

A total of 320 patients are the focus of this report. The median age of patients when hiccups were first reported in the medical record was 63 years (range: 21, 97 years) with 284 (89%) men and 36 (11%) women. The most common diagnose was gastrointestinal cancer. Hiccups most frequently occurred daily, as seen in 194 patients (62%), and the most common duration was less than 1 week, as seen in 146 patients (47%). However, nine patients had had daily hiccups for greater than 6 weeks, and 5 had symptoms for years. Cited etiology was non-chemotherapy medications in 36 (11%) and cancer chemotherapy in 19 (6%). Complications occurred in approximately a third and included insomnia in 51 patients (16%); hospitalization or emergency department visit in 34 (11%); and musculoskeletal pain in 23 (7%). Baclofen was the single most prescribed agent for hiccup palliation, but 100 patients received more than one medication. Medical procedures, which included acupuncture, paracentesis, or phrenic nerve block, were performed in 5 patients. In 234 patients (73%), the medical record documented hiccup cessation.

**Conclusions:**

Hiccups appear to be highly problematic in a small subset of patients with cancer with no well-defined palliative approaches.

## Introduction

An estimated 15–40% of patients with cancer experience hiccups [1–4]. Because single-patient case reports, case series, systematic reviews and meta-analyses, and data base studies comprise the predominant source of information on hiccups, the published literature appears skewed towards patients with more extreme hiccup symptomatology [4–6]. To date, this literature suggests that oncology drugs such as cisplatin, carboplatin, irinotecan, and dexamethasone cause hiccups and that the cancer itself with, for example, metastases to the diaphragm can also cause hiccups [7–9]. Furthermore, this drug- or tumor-induced adverse event appears often to result in sleep deprivation, poor oral intake, aspiration, and, at times, even death [5, 10]. Therapeutically, the Food and Drug Administration (FDA) has previously approved only one drug, chlorpromazine, for hiccup palliation, but the reports cited above point to other interventions, such as baclofen or gabapentin, that might also provide palliation [11–17].

To our knowledge, no previous study has attempted to investigate hiccups in patients with cancer with a design that allows for detailed patient-level data, as derived from a single-institution study. Similarly, to our knowledge, no prior study has attempted to strike a balance intended to capture both severe and less severe hiccup symptomatology by assessing patients with hiccups in a consecutive manner. In view of this potential for antithetic bias in the published literature, several questions remain unanswered. Who develops hiccups? In general, how severe are these hiccups? Currently, how are healthcare providers attempting to palliate hiccups, and how effective are these interventions? This multi-site, single institution, exploratory study was undertaken to begin to answer these questions.

## Methods

### Overview

The Mayo Clinic Institutional Review Board (IRB) provided approval to proceed with this study (#21–000354). This multi-site study from the Mayo Clinic relied on a broad catchment area that included Minnesota, Florida, Arizona, and surrounding states.

### Medical record ascertainment and data acquisition

The Mayo Clinic medical record system was interrogated to identify adult patients with cancer and with hiccups that had occurred between January 1, 2017 and December 31, 2020. The institutional medical record system allowed for generating large lists of patients by means of a search function with keywords, such as “hiccups” and “cancer,” and this approach was used here. The above relatively recent start date was chosen because it served to capture a contemporary experience of hiccups. Based on Minnesota law, patients who requested that their medical record not be used for research purposes were excluded (3% of all patients).

Medical records were reviewed consecutively and by hand to confirm that all patients were adults; had been diagnosed with cancer; and, during the designated interval, had experienced hiccups. A deliberate decision was made to include patients with skin cancers to capture as broad a spectrum of cancer patients as possible. Detailed data abstraction that recorded key patient demographics; baseline clinical characteristics; the frequency and duration of hiccups; and etiology, interventions, complications, and outcomes related to hiccups was undertaken. Additionally, information on whether cancer therapy was ongoing when hiccups were first reported was also abstracted. Although two investigators (YA and AR) performed most of this hand review, two other investigators (NAM and AJ) performed spot checks to confirm accuracy.

### Analyses

Data are presented with summary statistics that include medians and ranges and percentages. In view of the exploratory nature of this study, all data are presented descriptively. If a specific data item included missing data, this absence was denoted only if it exceeded > 3% of the initial data within that category.

## Results

### Demographics

A total of 320 patients are the focus of this report after 5 were excluded because of no cancer and 5 because of no hiccups.

The median age of patients when hiccups were first reported in the medical record was 63 years (range: 21, 97 years). Two hundred eighty-four patients (89%) were men, and 36 (11%) women. Slightly under 60% of patients were receiving active cancer therapy when hiccups were first reported. Other demographics, including height, which has been suggested to be directly associated with hiccups development; cancer type; and number of comorbidities also appear in Table 1. The top three most common cancer diagnoses were gastrointestinal, dermatologic, and hematologic, respectively. Notably, 132 patients (41%) had esophageal-related morbidity.

**Table 1:** Demographics (*n* = 320)

Median age in years (range)	63 (21, 97)
Gender (%)	
Male	284 (89)
Female	36 (11)
Height in centimeters (range)	177 (147, 199)
Cancer (%)^a^	
Gastrointestinal	109 (34)
Dermatologic	86 (27)
Hematologic	84 (26)
Genitourinary	79 (25)
Brain tumor	25 (8)
Head and neck cancer	16 (5)
Other^b^	38 (12)
Median number of co-morbidities (range)^c^	4 (0,16)

^a^Percentages do now sum to 100% because over 20% of patients had more than one cancer type

^b^Other includes sarcoma (13), gynecologic (8), lung (6), breast (6), endocrine (4), ocular (1)

^c^Esophageal-related morbidity was the most common

### Hiccup frequency, duration, etiology, and complications

Hiccups most frequently occurred daily, as seen in 194 patients (62%), and the most common duration was less than 1 week, as observed in 146 patients (47%) (Table 2). However, nine patients had had daily hiccups for greater than 6 weeks, and 5 of these patients had symptoms that persisted for years.

**Table 2:** Hiccup Characteristic (*n* = 320)^a^

Frequency of hiccups	
Daily	194 (62)
Once every few days	28 (9)
Less than the above categories	14 (4)
Cannot tell	84 (26)
Duration	
< 1 week	146 (47)
1–4 weeks	75 (24)
> 4 weeks but no more than 6 weeks	4 (1)
> 6 weeks	44 (14)
cannot tell	51 (16)
Cause of hiccups	
Esophageal disease	2 (0)
Cancer	7 (2)
Medication (other than chemotherapy)	36 (11)
Multifactorial	19 (6)
Cancer chemotherapy	19 (6)
Other^b^	33 (9)
Cannot tell	210 (66)
Complications from hiccups	
None	203 (63)
Sleep problems	51 (16)
Hospitalization or emergency department visit	34 (11)
Musculoskeletal pain	23 (7)
Nausea	21 (7)
Vomiting	21 (7)
Shortness of breath	15 (5)
Poor ortal intake	15 (5)
Anxiety/depression	4 (1)
Aspiration	2 (0)
Weight loss	3 (1)
Other^c^	15 (5)

^a^For causes and complications, percentages in parentheses do not sum to 100% because of patient overlap in categories

^b^Other includes nasogastric tube (5), abdominal distention (4), uremia (3), cerebrovascular accident (3), diaphragmatic irritation from cancer (2), radiation (2), stent migration (1), esophageal stent placement (1), tube feedings (1), choking on food (1), indigestion (1), encephalopathy (1), hiatal hernia (1), enteritis (1), splenomegaly (1), chest tube (1), percutaneous gastric tube (1), anesthesia (1), diaphragmatic irritation from pulmonary infiltrates, esophageal dilatation (1), hyponatremia (1)

^c^Other includes fatigue (3), dysphagia (3), choking (2), throat pain (2), difficulty talking (1), delay in radiation (1), hemoptysis (1), compromise in imaging study (1), altered taste (1)

In 210 patients (66%), no specific cause of the hiccups was cited. However, when a cause was cited, the etiology was most often traced to non-chemotherapy medications in 36 (11%) and cancer chemotherapy in 19 (6%) (Table 2). Interestingly, although prior case reports had not cited lisinopril as a cause of hiccups, one patient described it as such, noting that the hiccups started with the initiation of the drug and stopped upon stopping the drug.

In terms of complications, again, the majority, or 203 of 320 patients (63%) had none. However, 51 patients (16%) had sleep problems; 34 (11%) had a hospitalization or emergency department visit; 23 (7%) had musculoskeletal pain; and a variety of other complications were noted (Table 2).

### Therapeutic interventions and Outcomes

Most patients 239 (75%) managed hiccups on their own with no medical intervention (Table 3).

**Table 3:** Hiccup Interventions and Outcomes

Did the patient see a specialist for hiccups?	
No	285 (90)
Yes^a^	31 (9)
Cannot tell	5 (1)
Were the hiccups treated by a healthcare provider?	
Yes	239 (75)
No	72 (23)
Cannot tell	9 (2)
Treatment rendered (overlap was seen in categories):	
Medication^b^	239 (75)
Patient interventions^c^	26 (8)
Medical procedure^d^	5 (2)
Multiple	4 (1)
Did the hiccups stop?	
Yes	234 (73)
No or cannot tell	86 (27)

^a^The most frequently consulted specialist was a gastroenterologist (*n* = 10)

^b^The most common single-agent medications were baclofen (*n* = 62), chlorpromazine (*n* = 28), metoclopramide (*n* = 7), and gabapentin (*n* = 5). Many patients (*n* = 100) received more than one medication

^c^Patient interventions included drinking water (*n* = 7), holding their breath (*n* = 1), other (*n* = 14), and multiple (*n* = 4)

^d^Medical procedures included acupuncture (*n* = 3), paracentesis (1), and phrenic nerve block (*n* = 1)

However, when medical interventions were resorted to, the most frequent consisted of prescription medications with baclofen the most prescribed single agent. One hundred patients (31%) received more than one medication. Of those patients who received medications for hiccups, a small subset (*n* = 5) received a medical procedure, which included acupuncture, paracentesis, or phrenic nerve block. In 234 patients (73%), the medical record documented evidence of hiccup cessation, but it was often not clear whether the intervention was the true cause of hiccup cessation.

## Discussion

This study focused on hiccups in patients with cancer and found that most had short-term, limited symptomatology. However, over one-third of patients developed symptoms that lasted a week or longer, and a small subset had ongoing, frequent symptoms that continued for several weeks or longer. Furthermore, a third of patients develop hiccup-induced symptoms, such as insomnia and pain. Interestingly, to our knowledge, few earlier studies have reported on the extent to which patients access medical care for hiccup palliation, but here we observed that approximately one in 10 patients sought help in an emergency department or hospital for hiccup palliation. In contrast to earlier reports that tended heavily to emphasize the complications of hiccups, this study observed that hiccups are seemingly inconsequential in the majority but clearly troubling in a small subset [4, 10]. This hiccup-related morbidity – when coupled with the many other known challenges faced by patients with cancer – show that hiccups are highly problematic for a subgroup of patients with cancer.

Although hiccups appear to be trivialized because of their commonplace nature and self-limited course, the National Institute of Health (NIH) and the National Organization for Rare Disorders (NORD) have categorized intractable hiccups as a rare disease [18, 19]. The latter is a designation for illnesses or disease entities that affect fewer than 200,000 people at a snapshot in time. Although the current study was not designed to comment on the percentage of all cancer patients who develop hiccups, it does provide perspectives on hiccup-related morbidity, underscoring how this entity can lead to morbidity and an increased need to access medical care in a small but notable group of patients with cancer. Based on such morbidity, this study suggests that the NIH’s and NORD’s designation of labeling hiccups as a rare disease is merited and that hiccups in patients with cancer should not be summarily downplayed.

Interestingly, this study underscores the challenges of demonstrating the therapeutic efficacy of hiccup interventions. To date, only a small number of published randomized, controlled trials, each with fewer than 40 patients, have suggested that agents such as baclofen and metoclopramide might palliative hiccups; this paucity of research suggests that more such research should be conducted [13, 20–22]. Admittedly, in most patients, the hiccups eventually resolve on their own with no or little intervention; this study confirms the same. However, in other patients, as seen in this study, a variety of therapeutic agents have been employed, leaving open the possibility that the last intervention to be tried presumably prior to spontaneous resolution of hiccups might be falsely declared as effective. This aspect of spontaneous resolution, the rarity of persistent/chronic hiccups, and that fact that only one drug provides precedent for how to acquire FDA approval of a drug for hiccups highlight the challenges of studying hiccup palliation in a rigorous and definitive manner. Yet, the data presented here -- including the complications of sleep disturbance, emergency department visits and hospitalizations, musculoskeletal pain, and others – suggest the need to develop novel study designs for hiccup palliation and to thereby enable healthcare providers to have access to a broader and more effective armamentarium of interventions for managing hiccups.

Finally, this study has limitations, the most notable of which is its retrospective design, which carries inherent challenges, including reporting bias. Although we had sought to provide a greater degree of balance in the literature on hiccups, reporting bias is likely at work in the current study, too. Those patients with more problematic hiccups were perhaps more likely to voice their concerns to their healthcare providers, and healthcare providers were more likely to include such information in the medical record if the hiccups were more severe or problematic. It should be noted, however, that hiccups are likely trivialized not only among healthcare providers but also among patients. Thus, in effect, any study of hiccups is likely to show some degree of bias toward more severe symptomatology, if for no other reason, then because patients themselves forget to mention minor, transient symptoms. In this context, despite this potential bias, the current study succeeded in its goal of illustrating a full spectrum of hiccups symptoms in patients with cancer – from mild and transient to relatively severe and persistent. The current study also showed that this symptomatology can be burdensome and that patients’ needs are sometimes unmet.

## Data Availability

The datasets generated and/or analysed during the current study are not publicly available due to confidentiality but are available from the corresponding author on reasonable request.
